# Coupled Resonators for Sound Trapping and Absorption

**DOI:** 10.1038/s41598-018-32135-5

**Published:** 2018-09-14

**Authors:** Rasha Al Jahdali, Ying Wu

**Affiliations:** King Abdullah University of Science and Technology (KAUST), Division of Computer, Electrical and Mathematical Sciences and Engineering, Thuwal, 23955-6900 Saudi Arabia

## Abstract

The leakage of sound waves in a resonance based rainbow trapping device prevents the sound wave being trapped in a specific location. In this study, we report a design of sound trapping device based on coupled Helmholtz resonators, loaded to an air waveguide, which can effectively tackle the wave leakage issue. We show that coupled resonators structure can generate dips in the transmission spectrum by an analytical model derived from Newton’s second law and numerical analysis based on finite-element method. An effective medium theory is derived, which shows that coupled resonators cause a negative effective bulk modulus near the resonance frequency and induce flat bands that give rise to the confinement of the incoming wave inside the resonators. We compute the transmission spectra and band diagram from the effective medium theory, which are consistent with the simulation results. Trapping and high absorption of sound wave energy are demonstrated with our designed device.

## Introduction

A metamaterial usually possesses unusual material parameters, indicating it is capable of achieving exotic physical properties beyond naturally occurring materials, such as negative refraction. In acoustic metamaterial, engineering unit structures may give rise to anomalous material parameters at resonance frequencies^1^. Many of these structures can be characterized by classical mechanics. For example, Helmholtz resonators (HRs), a widely used element in the design of acoustic metamaterials, can be modeled by a classical mass-spring system: the compressible fluid inside the cavity acts as a spring, while the fluid in the neck acts as the mass. Fang *et al*.^2^ realized the resonance-induced negative effective bulk modulus for ultrasound by a waveguide connected to a series of HRs. Due to the frequency dispersion of the local resonances, negative bulk modulus was observed, which causes a flat band in dispersion relation. Thus, the group velocity goes to zero and slow sound phenomenon was observed.

Slowing down acoustic waves introduces a delay to the sound and allows temporary storage of sound waves in the resonator structure, resulting in strong coupled sound waves and leading to novel sound application such as super-absorption^3–6^ and rainbow trapping^7–9^. To demonstrate perfect acoustic rainbow trapping, strong acoustic dispersion by local resonances is desired. Many structures have been proposed to achieve a rainbow trapping effect, including chirped sonic crystal^10^, periodically grooved rigid metawire^11^, coiling-up space metamaterial^7,8^, waveguide composed of numerous phonon barriers^12^, phoxonic crystal slab with air holes drilled on solid membrane^13^, and an array of gradient grooves perforated on a rigid panel^9^. Meanwhile, considering the loss, rainbow trapping with perfect absorption is observed^4^.

To achieve perfect absorption, the impedance of the structure should match that of the background medium. However, it is in general difficult to satisfy such condition for airborne sound. Broadband perfect sound absorption has been realized by slow sound phenomena in closed^6,14–26^ and non-closed^3–5,27–31^ sub-wavelength waveguides. If the leakage of the energy and the inherent loss of the device at the resonance frequency are balanced, critical coupling between the system and the environment is achieved^32,33^. In particular, the problem of achieving perfect absorption in a non-closed waveguide, with the reflection and transmission taken into account, becomes complicated. This is because the scattering matrix exhibits two different eigenvalues that rely on the values of the transmission and reflection coefficients of the structure, and zero eigenvalues are required to turn the transmission and reflection into perfect absorption at the resonance frequency^5^. This is referred to as the critical coupling condition. By a careful design which fulfils the critical coupling condition, perfect sound absorption is demonstrated^29^. When the symmetric and antisymmetric modes are critically coupled, implying that the eigenvalues of the scattering matrix vanish, the incident wave is perfectly absorbed within the structure, and this is the underlying mechanism of the critical coupling condition. Experiments show that critical coupling condition can be satisfied by two HRs loaded to a waveguide and perfect absorption is realized at a specific frequency^5^. Unfortunately, simultaneous critical coupling of the symmetric and antisymmetric modes is not always attainable. Quasi-perfect absorption is observed by implementing strong sound dispersion, which occurs when many layers of N identical HRs are loaded to the waveguide, and the symmetric and antisymmetric modes are approaching in very close resonance frequency^3^. Recently, the same structure but with gradient geometry was designed to slow down the sound waves with different frequencies at different locations so that rainbow trapping and perfect absorption were realized^4^. Owing to the overlapping of the bandgaps of the neighboring resonators, however, the desired functionality of rainbow trapping at specific locations is not perfectly achieved.

In this work, we propose a new mechanism to slow down sound wave by coupling N HRs in one unit cell. This approach offers flexibility in engineering the bandgaps induced by the HRs because each HR causes a stop band. Trapping and broadband absorption are demonstrated. We derive analytic formula for the proposed design from the Newton’s second law. In addition, we developed an effective medium model by using the principle of the hidden source of volume. Trapping and absorption functionalities are demonstrated by finite-element simulations.

## Results

The physical configurations of N HRs coupled in one unit cell loaded to the waveguide can be considered as coupled N mass-spring system^34^. As a consequence, N resonances are generated and each band gap ascribed to one HR in which the width of the gap can be tuned by changing the volume of the cavity. This mechanism provides strong sound dispersion to slow wave propagation and to trap the sound wave at the precise location without leakage.

### Analytic formula

**Figure 1:**
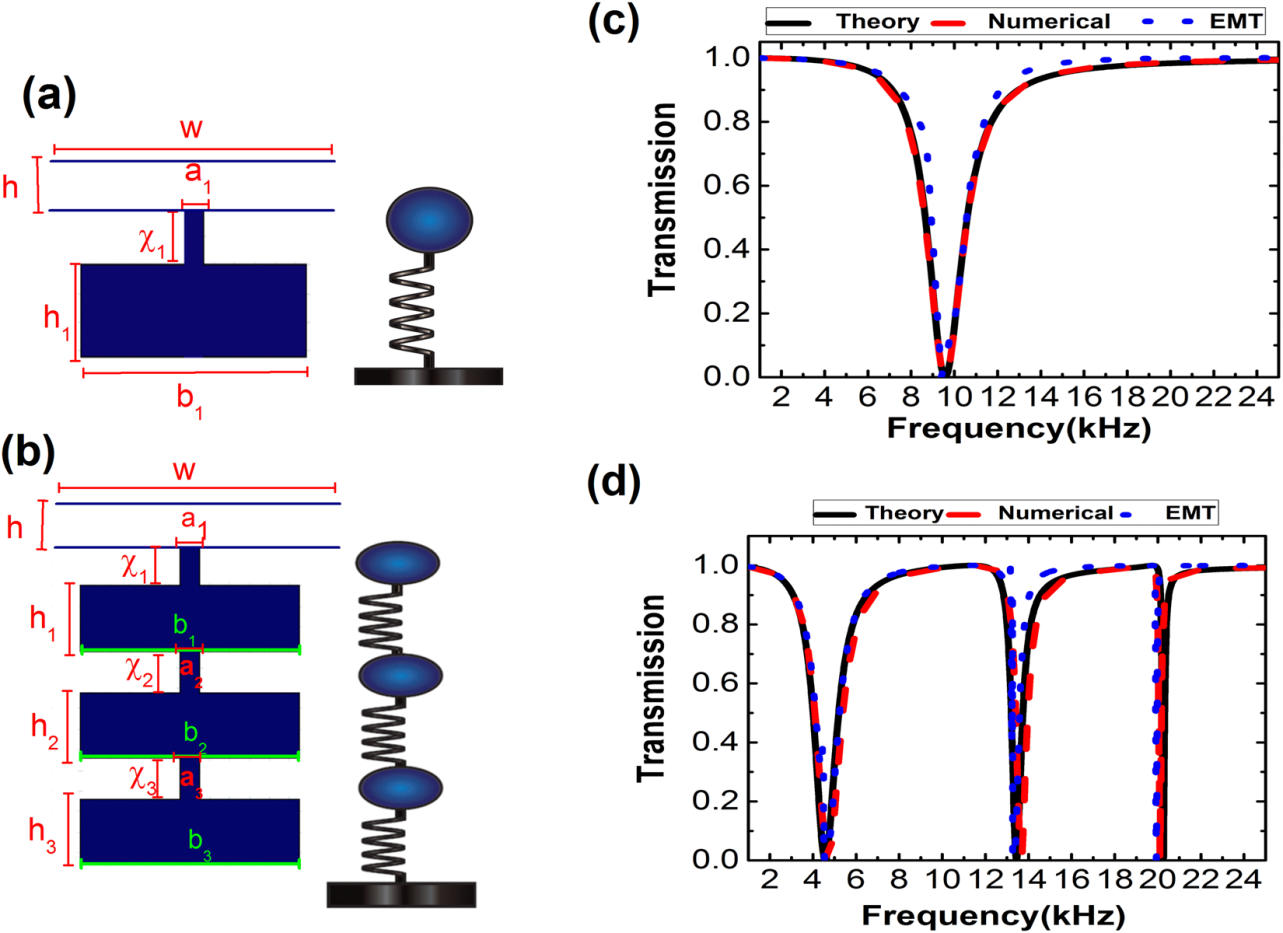
Schematic of the building block of (**a**) single HR and (**b**) three coupled HRs metamaterial, and the corresponding mechanical mass-spring system. (**c,d**) The transmission coefficient of a plane wave incidence from the left side on the unit cell theoretical prediction (black solid curve), and numerical simulation (red solid curve), compared with the transmission coefficient of the same plane wave incident on an effective homogenous slab (blue dots curve) for the unit cell is illustrated in (**a,b**), respectively.

We start with a single standard HR, composed of a cavity and a short neck on one side, loaded to a non-closed air waveguide, as illustrated in Fig. 1(a). The width and length of the short neck are denoted as ${a}_{1}$ and ${\chi }_{1}$, the width and length of the cavity are ${b}_{1}$ and ${h}_{1}$, the width and length of the waveguide are *w* and *h*. Consider the air in the neck, by employing Newton’s second law, we have the following expression:

1 $${\rho }_{0}{a}_{1}{{\chi }_{1}}^{^{\prime} }{\ddot{\eta }}_{1}={a}_{1}(p-{\rm{\Delta }}{p}_{1})$$

where ${\rho }_{0}$ is the mass density of air, ${\chi }_{i}^{^{\prime} }(={\chi }_{i}+{a}_{i}[(0.514-0.318\,\mathrm{ln}(k{a}_{i}))+{h}_{i}/3{b}_{i}+\sum _{n=1}({b}_{i}^{2}/{(n\pi )}^{3}{a}_{i}^{2}){\sin }^{2}$ $(n\pi {a}_{i}/{b}_{i})])$ is the effective length of the neck of the *i*^th^ resonator^35^, $p$ is the pressure that is applied to the resonator, and ${\eta }_{1}$ is the displacement of the air. ${\rm{\Delta }}{p}_{1}$ is the variation of the pressure inside the resonator, and is given by $-{\kappa }_{0}{a}_{1}({\eta }_{1}/{\nu }_{1})$ where ${\kappa }_{0}$ is the bulk modulus of air, and ${\nu }_{1}(\,=\,{b}_{1}{h}_{1})$ is the volume of the cavity. In what follows, we use the harmonic expression for the pressure and the displacement at angular frequency $\omega$, which take the following form $p=p{e}^{i\omega t}$ and ${\eta }_{1}={\eta }_{1}{e}^{i\omega t}$, respectively. Eq. (1) is converted into:

2 $${\eta }_{1}=-\,\frac{{a}_{1}p}{{\omega }^{2}{\rho }_{0}{a}_{1}{\chi ^{\prime} }_{1}+({a}_{1}^{2}{\kappa }_{0}/{\nu }_{1})}$$

The velocity of a particle ${u}_{1}$ is determined as ${u}_{1}={\eta ^{\prime} }_{1}=i\omega {\eta }_{1}$, which yields the normalized specific impedance of the resonator, and can be written in the form

3 $$\xi =\frac{p}{{\xi }_{0}{u}_{1}}=\frac{-1}{i\omega {\xi }_{0}}[{\omega }^{2}{\rho }_{0}{\chi ^{\prime} }_{1}+\frac{{\kappa }_{0}{a}_{1}}{{\nu }_{1}}]$$

The transmission coefficient of the system can be calculated from following relation^34^:

4 $$T=\frac{2h\xi }{(2h\xi +{a}_{1})}$$

The general case of the previous analysis can be demonstrated by increasing the number of the resonators in each unit cell attached to the waveguide. The transmission coefficient of N HRs can be calculated by applying the equation of motion for each resonator, which is written in the form

5 $${\rho }_{0}{a}_{i}{\chi }_{1}^{^{\prime} }{\ddot{\eta }}_{1}={a}_{i}({\rm{\Delta }}{p}_{i}-{\rm{\Delta }}{p}_{i-1}),\,i=2,\mathrm{...},N$$

The variation of the pressure inside the *i*^th^ resonator is expressed as ${\rm{\Delta }}{p}_{i}=(-{\kappa }_{0}/{\nu }_{i})({a}_{i}{\eta }_{i}-a{}_{i-1}\eta _{i-1})$ where ${\nu }_{i}$ $(\,=\,{b}_{i}{h}_{i})$ is the volume of the *i*^th^ cavity. Coupling these resonators leads to the following equation:

6 $${\boldsymbol{q}}(\omega ,{a}_{j},{\chi ^{\prime} }_{j},{h}_{j},{b}_{j}){\eta }_{j}-{\delta }_{1,j}(p{a}_{j=1}-{\kappa }_{0}{a}_{j=1}^{2})=0,\,j=1,\mathrm{...},N,$$

***q*** represents a tridiagonal stiffness matrix, where the subdiagonal and superdiagonal are given by $${{\boldsymbol{q}}}_{i,j-1}=-{\kappa }_{0}{a}_{j}{a}_{j-1}/{\nu }_{j-1}$$, $${{\boldsymbol{q}}}_{j,j+1}=-{\kappa }_{0}{a}_{j}{a}_{j+1}/{\nu }_{j+1}$$, and the main diagonal is expressed as $${{\boldsymbol{q}}}_{j,j}=-\,{\rho }_{0}{a}_{j}{\chi }_{j}^{^{\prime} }{\omega }^{2}+$$$${\kappa }_{0}{a}_{j}^{2}({\nu }_{j}^{-1}+{\nu }_{j-1}^{-1})$$. We consider a 3 × 3 matrix since the system is composed of 3 coupled HRs in a unit cell as shown in Fig. 1(b), and the displacement of the air in neck of the first resonator is obtained by:

7 $${\eta }_{1}={a}_{1}p(\frac{{q}_{23}{q}_{32}-{q}_{22}{q}_{33}}{{q}_{11}{q}_{23}{q}_{32}+{q}_{12}{q}_{21}{q}_{33}-{q}_{11}{q}_{22}{q}_{33}})$$

Then the normalized specific impedance of three coupled resonators is given by:

8 $$\xi =\frac{p}{{\xi }_{0}{u}_{1}}=\frac{-1}{i\omega {a}_{1}{\xi }_{0}}[\frac{{q}_{11}{q}_{23}{q}_{32}+{q}_{12}{q}_{21}{q}_{33}-{q}_{11}{q}_{22}{q}_{33}}{{q}_{23}{q}_{32}-{q}_{22}{q}_{33}}]$$

Substituting $\xi$ into Eq. (4), we obtain the transmission coefficient of the unit. To verify the theory and give a clear physical picture, a numerical simulation was carried out using COMSOL Multiphysics. Figure 1(c,d) show the transmission coefficient for two structures with different numbers of resonators, indicating the number of transmission dips is equal to the number of resonators. The red solid curve represents the simulated transmission spectrum of a plane wave normally incident from the left of the waveguide, where the black solid curve is obtained from Eq. (4). The geometric parameters used for the single HR case are ${a}_{1}=0.5[mm]$, ${\chi }_{1}=1\,[\mathrm{mm}]$, ${h}_{1}=1.6\,[\mathrm{mm}]$, ${b}_{1}=5.5\,[\mathrm{mm}]$, and with $h=4\,[\mathrm{mm}]$ and $w=7\,[\mathrm{mm}]$. For triply coupled HRs case, each resonator is identical to the single HR described here. Good agreement between the theoretical prediction and numerical simulation is seen from Fig. 1(c,d).

### Effective medium theory

We developed an effective medium theory based on the principle of the hidden source of volume^36,37^. The hidden source of volume for a single resonator or multiple resonators can be obtained by ${\rm{\Delta }}{\phi }_{h}={a}_{1}{\eta }_{1}$. Therefore, the effective bulk modulus of single resonator and coupled resonators system can be rewritten as

9 $${\kappa }_{eff}={\kappa }_{0}(1+\frac{{\rm{\Delta }}{\phi }_{h}}{{\rm{\Delta }}\phi })$$

${\rm{\Delta }}\phi$ represents the change in waveguide volume. Using Eq. (9), we write ${\kappa }_{eff}$ for a single resonator as

10 $${\kappa }_{eff}=\frac{{\kappa }_{0}}{1+\frac{{a}_{1}^{2}{\kappa }_{0}}{v}(\frac{{\kappa }_{0}{a}_{1}}{{v}_{1}}-{\omega }^{2}{\rho }_{0}{\chi ^{\prime} }_{1})}$$

where $v(\,=\,w\times h)$ denotes the volume of the waveguide, and ${\kappa }_{eff}$ for three coupled resonators as

11 $${\kappa }_{eff}=\frac{{\kappa }_{0}}{1+\frac{{a}_{1}^{2}{\kappa }_{0}}{v}(\frac{{q}_{23}{q}_{32}-{q}_{22}{q}_{33}}{{q}_{11}{q}_{23}{q}_{32}+{q}_{12}{q}_{21}{q}_{33}-{q}_{11}{q}_{22}{q}_{33}})}$$

**Figure 2:**
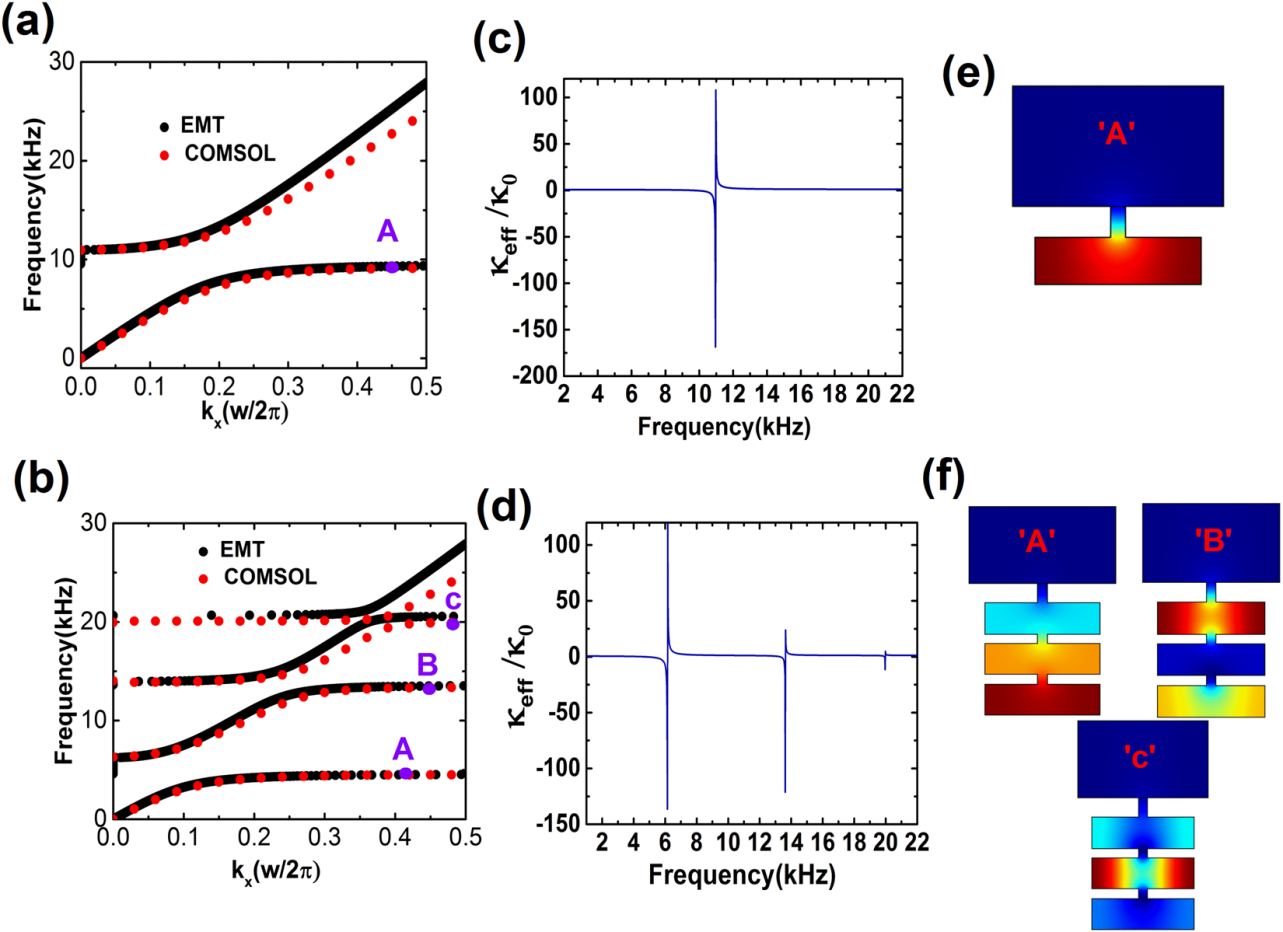
The verification of the derived EMT. Band structure calculations of (**a**) single HR and (**b**) three coupled HRs using COMSOL (red dots), compared with EMT (black dots). (**c,d**) Corresponding effective bulk modulus calculated from Eqs (10 and 11). Eigenfield distribution for points (**e**)“A” marked in (**a**), (**f**) “A”, “B”, and “C” marked in (**b**). Dark blue and dark red represent the zero and maximum values of the pressure intensity.

The transmission spectra of slabs with effective moduli given by Eqs (10) and (11) and effective mass density of ${\rho }_{0}$, are presented in dotted blue curves in Fig. 1(c,d). Good agreements among the theory, numerical simulation and the effective medium prediction are observed. These results verify the effective medium prediction and indicate that the dips in transmission spectra correspond to the coupling effect of the resonators, and hence the sound energy is strongly localized in the resonators at the resonance frequencies where the coupling between each resonator determines the resonance frequency. We also compute the band structure of a single HR and a triply coupled HRs using COMSOL and plot the results in red dots as illustrated in Fig. 2(a,b). For comparison, the band structures predicted by the EMT are plotted in solid black curves. Figure 2(c,d) show the corresponding effective bulk modulus. Typical resonance behaviors are observed and the more the HR, the more the resonances. The occurrence of negative modulus is attributed to the instantaneous displacement of the mass in each resonator flips the phase from in-phase to out-of-phase at the resonance frequencies^2^. To further comprehend the mechanism, we plot the eigenfield patterns at points labeled on the band structure at the flat branch in Fig. 2(e,f). It should be noted from the field distribution at each eigenfrequency in the flat band that the pressure intensity is confined in the unit cell with a high concentration of pressure intensity on one resonator, and owing to the coupling effect of the resonators the pressure intensity spreads out significantly into other resonators. Here, we would like to point out that the classical mass-spring model is based on lumped element method, which requires the dimensions of the structure must be sub-wavelength. In Fig. 2(f), the wavelength at the resonance frequencies ($4.5\,[\mathrm{kHz}]$, $13.5\,[\mathrm{kHz}]$, and $20\,[\mathrm{kHz}]$) is about 9.8, 3.3, and 2.2 times the total length of the unit cell, respectively. The pressure distribution inside the cavities at the second and third resonance frequencies is not uniform, indicating the higher order modes. However, the lumped element approximation is still valid for higher order modes since the length of the structure is smaller than the wavelength at resonance frequencies. The calculations of band structure and transmission spectra verify our conclusion, as demonstrated in Figs 1(c,d) and 2(a,b). These results provide us with a clear evidence that by adding a resonator to the unit cell, a new flat band is attainable, and hence the group velocity goes to zero as well. Based on this mechanism, it is easy to see that the width and the number of the band gap with flat branches can be tuned by changing the number and the size of the resonators at desired frequency domains to achieve trapping effects.

### Acoustic trapping and absorption

**Figure 3:**
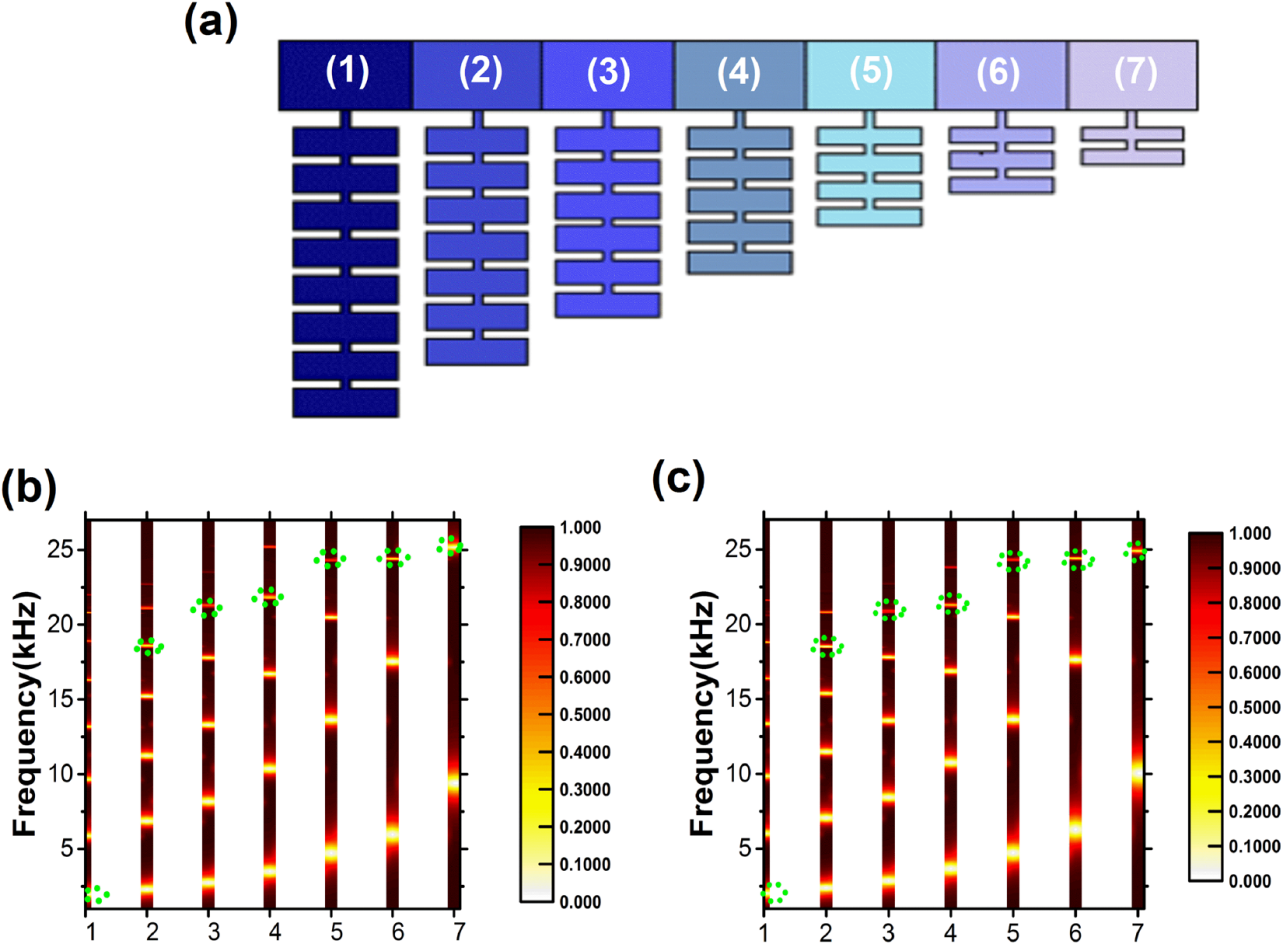
(**a**) Conceptual design of the trapping device, composed of air waveguide attached with 7 unit cells of coupled HRs with various numbers and sizes of cavities of the resonators. The contour plot of the transmission spectrum is calculated by (**b**) theoretical prediction and (**c**) numerical simulation for each unit illustrated in Fig. 3(a). Green dash ovals indicate the resonance frequency of the perfect sound trapping of each unit.

**Figure 4:**
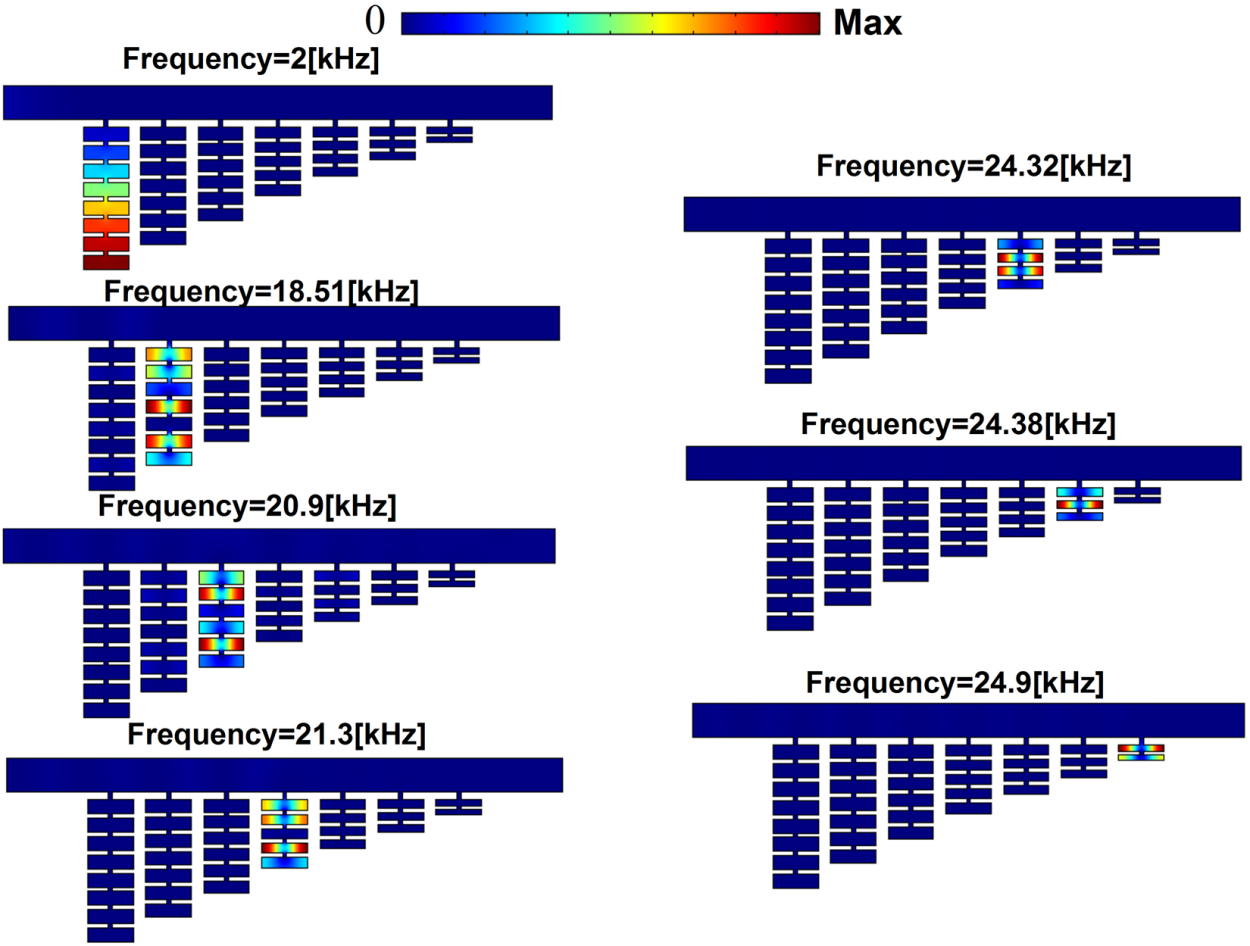
The pressure intensity at the resonances frequency marked in Fig. 3 ($2\,[\text{kHz}]$, $18.51\,[\text{kHz}]$, $20.9\,[\text{kHz}]$, $21.3\,[\text{kHz}]$, $24.32\,[\text{kHz}]$, $24.38\,[\text{kHz}]$, and $24.9\,[\text{kHz}]$) for units (1, 2, 3, 4, 5, 6 and 7), respectively.

From previous analysis, we noticed that coupled HRs offer more degrees of freedom, such as the number and the size of the resonators, in designing the acoustic trapping devices. Figure 3(a) shows an example of an acoustic metamaterial with different numbers of coupled HRs attached to an air waveguide. In this design, we consider 7 unit cells loaded to the air waveguide where an incident plane wave is coming from the left. To obtain excellent trapping effect, a gradient size of the cavities of the resonators in different unit cells has been used, whereas the size of the necks are fixed for all the HRs as demonstrated in Fig. 3(a). For simplicity but without loss of generality, identical resonators in one building block are utilized. ${a}_{j}$, ${\chi }_{j}$, ${b}_{j}$, and ${h}_{j}$ respectively correspond to the width and length of the short neck, and the width and length of the cavity of the *j*^th^ unit cell. We optimize the geometric parameters by gradually decreasing the number and the size of the resonators in different unit cells to obtain the trapping effect. The values of the geometric parameters are given in Table 1. The transmission spectra for the seven units are calculated *separately* and plotted collectively in Fig. 3(b) (theoretically results) and 3(c) (numerical results). The dark area indicates high transmission while bright regions correspond to low transmission. It is worth mentioning that there are multiple low frequency resonances but they are not suitable for precise trapping proposal. The sound wave energy will leak out rather than being trapped in the desired unit, due to the couplings between those low frequency resonance modes. However, for resonances at high frequencies, the Q-factor is high, and the coupling between neighboring modes are weak, leading to perfect sound trapping. The frequencies for sound wave trapping by each unit are chosen and indicated by green dashed ovals. It should be noted that although the first bandgap of one unit is much wider than the others, it overlaps with the first band gaps of its neighboring units and thus the coupling between them is unavoidable so the wave will not be trapped in the desired unit. Indeed, those first band gaps can be obtained by utilizing a single HR, which is not a good candidate to realize perfect sound trapping^4,31^. In this study, we provide an optimal solution via coupled HRs, producing stopbands with various widths, many of which have flat band edges and do not interact with band gaps of their neighboring units. Therefore, the waves are trapped in the desired unit rather than leak out. To examine the performance of our design, we measure the pressure intensity for each unit cell at the resonances marked in Fig. (4). The trapping effect is manifested by obviously enhanced pressure intensity in the desired unit cell (1, 2, 3, 4, 5, 6, and 7) at the resonance frequencies ($2\,[\text{kHz}]$, $18.51\,[\text{kHz}]$, $20.9\,[\text{kHz}]$, $21.3\,[\text{kHz}]$, $24.32\,[\text{kHz}]$,$24.38\,[\text{kHz}]$, and $24.9\,[\text{kHz}]$) that are predicted from theory, numerical analysis, and EMT. Since we do not consider loss here, the sound wave will eventually be reflected back^7,9,10^, despite the fact that the group velocity at the resonance frequencies is zero.

**Table 1:** Geometrical parameters for trapping device.

*j*	*a*_*j*_[mm]	*x*_*j*_[mm]	*h*_*j*_[mm]	*b*_*j*_[mm]
1	0.5	1	1.66	5.5
2	0.5	1	1.54	5.5
3	0.5	1	1.42	5.5
4	0.5	1	1.20	5.5
5	0.5	1	1.06	5.5
6	0.5	1	0.94	5.5
7	0.5	1	0.65	5.5

**Figure 5:**
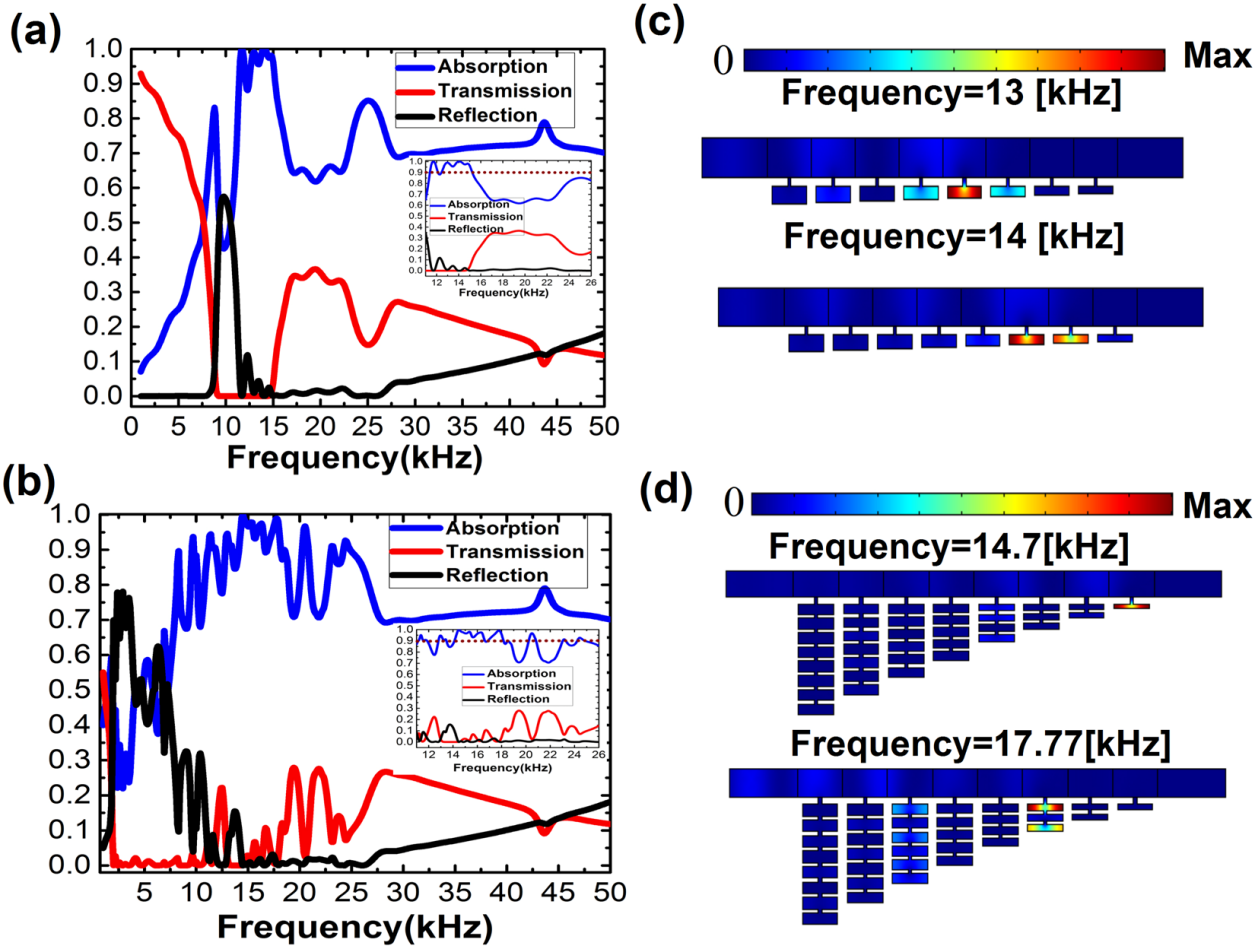
Simulated absorption, transmission, and reflection spectrum with the considered loss for (**a**) a single resonator and (**b**) coupled resonators in each unit. (**c,d**) The pressure intensity distribution at different frequency resonances for both designs, respectively.

One purpose of trapping is to achieve perfect absorption of energy over a broad frequency range from the slow sound propagation. We redesigned our proposed acoustic metamaterial by increasing the number of unit cells to 8, which is the same structure as the trapping device but we just added one more unit cell with a single HR, to achieve better results since more resonators lead to more peaks in the absorption spectrum, where the geometric parameters are given in Table 2. Here, we introduce thermo-viscous losses into the device. The loss is introduce by adding an imaginary part into the wave number $k=2\pi /{\lambda }_{0}-i\tau$ where ${\lambda }_{0}$ is the sound wavelength, and $\tau$ is the attenuation coefficient. In our model, the attenuation coefficient is given by $2\pi f/{c}_{0}\times \eta$, where $\eta$ represents the loss in air. Here, we set $\eta =0.01$. This is the convention adopted in the literature^7^. Figure 5(b) exhibits broadband and prefect energy absorption of coupled HRs metamaterial calculated by finite-element simulations. Over 90% of incoming sound energy are absorbed over a broad frequency range from $14.28\,[\mathrm{kHz}]$ to $18.02\,[\mathrm{kHz}]$, and perfect absorption is observed over the frequency range from $14.44\,[\mathrm{kHz}]$ to $14.55\,[\mathrm{kHz}]$ and from $17.62\,[\mathrm{kHz}]$ to $17.85\,[\mathrm{mm}]$. In Fig. 5(d), we also plot the pressure intensity distribution at various wavelengths of $23.3\,[\mathrm{mm}]$ and $19.3\,[\mathrm{mm}]$ ($14.7\,[\mathrm{kHz}]$, and $17.77\,[\mathrm{kHz}]$), showing that the sound waves are trapped and absorbed at different locations. For comparison, we calculate the reflection, absorption and transmission spectra of the same structure but only keep one HR in each unit. The results are plotted in Fig. 5(a). High absorption is also observed in a slightly narrower frequency range (from $12.7[\mathrm{kHz}]$ to $15\,[\mathrm{kHz}]$) compared to the coupled one. The sound pressure intensity distributions of different wavelengths are plotted in Fig. 5(c), which shows the sound waves are trapped in many units rather than a single unit.

**Table 2:** Geometrical parameters for absorption device.

*j*	*a*_*j*_[mm]	*x*_*j*_[mm]	*h*_*j*_[mm]	*b*_*j*_[mm]
1	0.5	1	1.66	5.5
2	0.5	1	1.54	5.5
3	0.5	1	1.42	5.5
4	0.5	1	1.30	5.5
5	0.5	1	1.06	5.5
6	0.5	1	0.94	5.5
7	0.5	1	0.82	5.5
8	0.5	1	0.70	5.5

## Discussion

Based on the coupled mass-spring model, we propose a design for acoustic metamaterial based on coupled HRs to trap sound waves with different frequencies at different locations and to absorb broadband sound wave energy. The study reveals that the coupling of HRs modes generates dips in the transmission spectrum, and more dips appear when more resonators are included in each unit cell. We also find in the band structure diagram that the flat bands at the resonance frequencies, which correspond to slow sound wave propagation with small group velocities, are induced by the negative value of the effective modulus that can be excited by the coupling of the resonators’ modes. Such a design, supporting and enhancing a slow sound resonance with extremely strong sound dispersion, is able to perfectly trap sound waves at the desired location rather than allow them to leak out. If the thermo-viscous loss effect is considered in the device, high and broadband sound absorption is achieved. Our findings may have prospective applications in acoustic device design such as acoustic insulation, acoustic filters, and broadband perfect absorbers.
